# Development of a National Certification Board Reboot and Readiness Course

**DOI:** 10.1111/jmwh.70022

**Published:** 2025-09-05

**Authors:** Melicia Escobar, Caitlin Givens, Katie DePalma, Heather M. Bradford

**Affiliations:** ^1^ Georgetown University Berkley School of Nursing, Nurse‐Midwifery/Women's Health Nurse Practitioner & Women's Health Nurse Practitioner Programs Washington District of Columbia

**Keywords:** academic performance, distance education, health workforce, midwifery, nursing, test‐taking skills

## Abstract

Pass rates for the American Midwifery Certification Board (AMCB) national certification examination (NCE) are declining. Accreditation Commission for Midwifery Education–accredited midwifery education programs are challenged to meet institutional pass rate goals and support graduates seeking to enter the midwifery workforce. There are financial, emotional, and social consequences for graduates who do not pass the AMCB NCE that undermine their success. A large, distance‐based nurse‐midwifery/women's health nurse practitioner program is successfully improving its AMCB NCE pass rates via an innovative, structured, and tuition‐free Boards Reboot & Readiness course. Designed for at‐risk students and graduates, the course emphasizes a trauma‐informed approach, focusing on self‐reflection, community building, content review, and high‐intensity faculty support. Student feedback and outcomes have informed iterative enhancements and revisions.

## INTRODUCTION

Graduates of Accreditation Commission for Midwifery Education (ACME)‐accredited midwifery education programs must successfully pass the American Midwifery Certification Board (AMCB) national certification examination (NCE) to become certified nurse‐midwives (CNMs) or certified midwives. Although pass rates on NCEs vary among health care professions,^1^, ^2^, ^3^ AMCB NCE overall pass rates have decreased nationally from 91.8% in 2015 (n = 610) to 73.0% (n = 1037) in 2024.^4^ The most significant drop occurred in 2018 (from 90.5% in 2017 to 80.8% in 2018) when the NCE was revised based on the 2017 Task Analysis.^5^ ACME requires accredited midwifery programs to report board pass rates within one year of graduation on an annual basis.^6^ Per ACME, midwifery programs may set their own institutional pass rate goal, and if they do not meet this goal, they must develop an improvement plan.

Beginning in 2019, AMCB pass rates decreased at one large, distance‐based online dual nurse‐midwifery (NM)/women's health nurse practitioner (WHNP) program with students in 49 states, consistent with national trends. Following the COVID‐19 pandemic in 2021, pass rates declined again. Although the challenge of preparing students to pass board certification examinations is not unique to nursing or midwifery,^7^, ^8^, ^9^ there is limited evidence for a predominant educational strategy to promote success on board examinations, before and after failure.^10^ A review of literature revealed no published strategies for improving AMCB board pass rates.

  

Continuing education (CE) is available for this article. To obtain CE online, please visit http://www.jmwhce.org. A CE form that includes the test questions is available in the print edition of this issue.

Failure to pass the AMCB NCE has a financial, emotional, and social burden. Each examination attempt costs $500. After a failed initial attempt, a retake may occur no sooner than 30 days; subsequent attempts require waiting 90 days.^11^ Each waiting period further delays entry to practice with correlated financial implications. In a 2022 national survey of midwifery students (n = 253) regarding graduate school financial support, the majority (62%) did not receive scholarships or loan repayment and thus rely on loans, which must be paid back after graduation.^12^ These financial hardships disproportionately impact graduates with pending graduate loan repayment or those of lower socioeconomic status. Many of the program's graduates return to full‐time nursing roles to pay their loans while waiting to initiate their first midwifery position.

Emotionally, the mounting student loans and financial obligations are stressful. Graduates can experience anxiety and depression after an examination failure, especially after the completion of a rigorous graduate program.^10^, ^13^ Failure can also undermine their confidence and sense of self‐worth.^14^ Socially, it can be challenging to communicate with fellow graduates who have passed the examination, leading to isolation.^14^ Graduates may experience shame in sharing their challenges with program faculty, family, friends, and future employers,^10^ leading to further isolation. The increased emotional challenges are traumatizing and can undermine study efforts and subsequent examination retake attempts.

**QUICK POINTS**

✦ With declining national pass rates for the American Midwifery Certification Board (AMCB) examination since 2015, some students at one nurse‐midwifery education program were determined to be at an increased risk of AMCB examination failure.
✦ A 7‐week online innovative and tuition‐free board certification preparation course was developed, which includes asynchronous and synchronous curriculum, access to a faculty‐developed multiple‐choice test bank, self‐reflection assignments, and high‐intensity faculty support.
✦ By applying trauma‐informed strategies, a board preparation course mitigates the impact of systemic racism in midwifery education and promotes a more equitable student learning environment.
✦ Faculty can promote student confidence and examination readiness through a holistic approach to content review, test‐taking strategies, time management tips, and the creation of a brave and safe space for learning.
✦ The use of a board certification preparation course is a highly effective approach to increasing board pass rates in one graduate midwifery education program and growing the midwifery workforce.

## Evolution of Structured NCE Support

In 2019, the program offered an informal, time‐intensive, one‐on‐one content review via video conferencing by one faculty member to each graduate who reported an AMCB examination failure. This evolved into a structured bimonthly small group review, ranging from 1 to 5 graduates using questions from the *Midwifery and Women's Health Nurse Practitioner Board Certification Review Guide* ^15^ as the primary review resource. This led to the faculty's examination of root causes of failure, which yielded 2 key findings: (1) students experience shame in the wake of failure, and (2) suboptimal performance (<80%) on the program's comprehensive exit examination was noted among graduates who had not passed AMCB. As a result, the review approach was adjusted to provide one‐to‐one or small group support with shame‐free emphasis and expanded to include graduates who scored <80% on their comprehensive exit examination.

Subsequently, the COVID‐19 pandemic revealed a third group of students at risk for examination failure. Students who experienced a delay in their clinical coursework (and thus program completion) due to a lack of clinical placement were less likely to pass the NCE. These students also reported feelings of isolation, as they were now disconnected from board examination preparation with their cohort. Altogether, with the identification of these additional at‐risk graduates, a a structured, shame‐free, and more time‐efficient approach was developed.

## TENETS OF THE COURSE: DESIGN AND IMPLEMENTATION

In 2024, program leadership developed and implemented the Boards Reboot & Readiness (BRR) course to promote graduates’ confidence as they either prepare for a second attempt at the NCE (ie, *reboot*) or enhance their board readiness for a first attempt. Although delivered as a 2‐credit course, BRR is offered tuition‐free.

### Eligibility and Enrollment

Faculty identified and invited 3 select groups of students eligible to enroll in the BRR course: (1) students who are completing their final (*integration*) clinical rotation later than expected and thereby several months removed from didactic coursework, (2) graduates who failed their comprehensive examination (scored <80%), and (3) graduates who did not pass the AMCB NCE on the first or second attempt. All integration students received messaging about the BRR course before the comprehensive examination. Faculty sent targeted informational emails to graduates whose certification status cannot be verified via the AMCB website.^16^ Because of the standard protections in place, AMCB does not disclose to education programs the names of graduates who do not pass. Thus, faculty extend invitations to the BRR course for the third group only if they self‐reported an unsuccessful attempt, limiting the opportunity to offer the course to all eligible graduates. Faculty try to foster a welcoming and shame‐free academic culture so students feel comfortable disclosing. Per institutional policy, faculty cannot email graduates with direct inquiries regarding their NCE outcomes.

### Curriculum Overview

Faculty designed the BRR course as a one‐year pilot that required financial and administrative support from nursing school leadership and curricular support from program faculty. The program offers the course 3 times a year as a 2‐hour, 7‐week live synchronous class using video conferencing. Class size ranges from 5 to 12 students each term. The curriculum focuses on different content areas each week based on the AMCB NCE blueprint.^11^ Because program graduates are eligible to be dually certified as CNMs and WHNPs, course faculty also review WHNP National Certification Corporation (NCC)‐specific content areas, including health assessment, diagnostic testing, pharmacology, and reproductive health care for individuals assigned male at birth. The course provides asynchronous and synchronous resources, drawing upon content presented in previous courses. In each synchronous session, course faculty reinforce asynchronous learning activities and provide content review, test‐taking strategies, individualized support and encouragement, and opportunities for peer connection. BRR students are typically juggling studying, registered nurse work, family obligations, and, for some, clinical hours. Consequently, faculty strongly encourage completing all assignments, although they are neither required nor graded. The BRR course assumes student self‐motivation and personal accountability.

### Asynchronous Curriculum

One week before class, faculty assign 3 tasks: (1) a BRR readiness reflection (Table 1), (2) an individualized study plan (Table 1), and (3) steps to study group formation. These tasks prompt course readiness and preserve synchronous time for content review and test‐taking strategies. The reflection assignment requires students to consider potential barriers and facilitators to studying and reviewing their comprehensive examination results, and, as applicable, their NCE results for strengths and weaknesses by content area. An individualized study plan details their daily activities and correlates time allotment with related considerations. To facilitate efficient and focused studying, faculty suggest using the Pomodoro Technique, a time management method that, for 1 to 4 hours, recommends studying in a series of 25‐minute blocks, known as *pomodoros*, each followed by a 5‐minute break period.^17^ This method requires students to turn off all potential distractions (eg, mobile devices and email). Finally, forming a study group is strongly encouraged for peer support and accountability. Study groups should consist of 2 to 3 people and meet weekly for at least an hour using video conferencing. Study groups are highly effective because they help students stay accountable and foster a motivating, supportive learning environment.^18^, ^19^

**Table 1:** Boards Reboot & Readiness Student Self‐Reflection Prompts

Pre‐BRR Reflections Questions	Considerations for Developing an Individualized Study Plan	Post‐BRR Reflection Questions
How did you prepare for your integration comprehensive examination (starting with the beginning of the term)? Does anything stand out to you as a risk factor for not passing your board certification examination (eg, personal health concerns, mental health concerns, time availability, knowledge gaps, motivation)? Since taking your integration comprehensive examination, have you identified any test‐taking challenges for yourself? What resources can you access during your studying phase to set you up for success? Consider study accountability groups, family and friends who can provide emotional support, and wellness activities (ie, exercise, yoga, meditation, counseling).	When in the day are you most alert and focused? How do you use dedicated study time? What are your self‐care activities?	What went well with your individualized study plan during these past 7 weeks and what could have gone better? Does anything stand out to you as a risk factor for not passing your board certification examination (eg, personal health concerns, mental health concerns, time availability, knowledge gaps, motivation)? What additional resources can you access during this last portion of your studying phase to set you up for success?

Abbreviation: BRR, Boards Reboot & Readiness.

In preparation for each synchronous session, students complete 100 multiple‐choice (MC) questions based on the weekly topic(s)using a faculty‐developed online test bank^20^ or the Nagtalon‐Ramos and Escobar^21^ certification review guide text. Reviewing MC questions is an effective study technique, as it serves as an active and engaged learning process rather than passively absorbing information from reading text. It also assists with information recall.^22^ If a question is answered incorrectly, faculty encourage students to review the topic via prior coursework or directly from a referenced resource. If they are unclear in understanding the content or the answer, unclear topics can be added to a “muddy points” collaborative online document. In part, BRR faculty use this document to inform content review in the next synchronous session. With awareness of students’ muddy points, BRR faculty have ample time to review before class.

At the end of the 7‐week course, students complete a final reflective assignment (Table 1) that prompts consideration of their participation in the BRR course, persisting facilitators and barriers to passing the examination, available resources, and wellness activities. Students can use this reflection to plan when to schedule their examination. Faculty strongly encourage students to be realistic and reflective about their life commitments and family stressors, focusing on their mental well‐being. For example, faculty have learned that certain factors can increase the risk for examination failure, including the birth of a child, planning a wedding, full‐time work obligations, or family obligations such as caring for an ill family member. Thus, faculty advise students to schedule their examinations when they are free from major distractions.

### Synchronous Curriculum

The faculty approach to each BRR class is characterized by setting a shame‐free, inclusive tone for the virtual classroom. The first class provides a course overview and review of success strategies, including test‐taking tips and time management strategies (Table 2). The group establishes community agreements to create a welcoming space where students can share challenges and celebrate progress with studying and examination readiness. The faculty invite students to share their studying and preparation experiences thus far.

**Table 2:** Test‐Taking Strategies

Strategy	Consideration
Slow down	Do not rush to complete the examination due to anxiety and fear of not finishing on time
Eliminate distractors	Before reading the answer choices, determine what the question is actually asking and hypothesize what the answer could be
Identify key words in the question	Examples include: never, not, none; all, always; only; except
Choose an answer based on the scenario presented	Avoid adding additional clinical information to the question Avoid applying elements to scenarios experienced in clinical practicums
Choose the answer based on the advanced practice nursing role	Avoid answering questions based on the registered nursing role
Stay anchored in content from leading midwifery textbooks rather than precepted clinical experiences alone	The leading midwifery textbooks are the primary resources used to write AMCB NCE questions Clinical practice varies based on preceptors, practice setting, and location and may not be aligned with current evidence
Skip difficult questions and return later	Returning to unanswered questions at the end of the examination is more time‐efficient than answering each one in turn Perseverating over difficult questions leads to mental exhaustion and wastes time that can be used to answer easier questions

Abbreviations: AMCB, American Midwifery Certification Board; NCE, national certification examination.

Beginning in the second week, faculty ground sessions in student reflection, sharing study progress, and mutual support. Next, faculty review the muddy points and provide study resources on the topic(s). Most of the class is dedicated to content review with test‐taking strategies infused via a series of 10 to 20 MC questions via PowerPoint (1 slide per question). Students submit their answer choice within several minutes using a video conferencing poll feature before the faculty shares the correct answer. Students discuss why they chose their answer, prompting self‐reflection and sharing of knowledge acquisition. When students choose incorrect answers, faculty also promote student self‐reflection. Common reasons include (1) reading too quickly or missing a keyword, (2) adding peripheral clinical information not provided in the question, (3) changing the answer choice at the last minute, or (4) a knowledge gap. Faculty discuss rationales for the correct answer and the incorrect distractors, allocating 5 to 10 minutes per question. A reference for each question provides validation and leads to a deeper review. When relevant, faculty review content summary slides. Synchronous sessions are not recorded to encourage student attendance and participation.

The program faculty wrote MC questions that were mapped to the AMCB and NCC WHNP NCE blueprints. Furthermore, question types are proportionate to the AMCB NCE format (approximately two‐thirds cover normal findings and one‐third cover abnormal findings; two‐thirds test clinical judgement and one‐third test knowledge).^11^ Several faculty have extensive experience writing MC questions through professional development activities, a faculty‐developed online test bank, and participation in a regional midwifery education program consortium focused on writing the comprehensive examination.

After completing the 7‐week course, students take a 175‐MC mock NCE. Despite students’ requests to rush the timeline, faculty intentionally do not offer the mock examination earlier, promoting BRR course completion and a thorough content review. Faculty offer individualized review sessions of the mock NCE to identify persistent test‐taking challenges and knowledge gaps.

### Applying Trauma‐Informed Pedagogy to Promote Equity

BRR faculty applied several trauma‐informed strategies throughout the curriculum design to foster a more accessible, inclusive, and equitable learning environment.^23^ In its most basic form, equity is everyone having access to what they need.^24^ Systemic racism and other forms of discrimination, including ability, body size, ethnicity, culture, country of origin, and gender within US midwifery education, have perpetuated inequitable learning environments for all students.^25^ Although other midwifery board examination preparation courses are on the market, some graduates do not have the financial privilege or social support to access them. With this in mind and grounded in the institutional Jesuit value of *cura personalis* (care of the whole person), faculty recognize that some students require more substantive support and a no‐cost opportunity.

Pedagogically, BRR faculty acknowledge students’ pervasive challenges during their midwifery education program that may inhibit success, many of which are disparately experienced by those from historically oppressed backgrounds.^26^ BRR students commonly shared these challenges with faculty (Table 3). Many students rely on their resilience to overcome obstacles and succeed. Thus, faculty adopted a trauma‐informed approach that does not require student disclosure of personal, financial, or social disadvantages. Instead, BRR faculty assume that all students have experienced hardship and trust in their resilience. Incorporating trauma‐informed strategies into the course design, including a detailed week‐by‐week syllabus, predictable synchronous session agendas, self‐reflection, peer support, and empathetic culture, promotes inclusion and equity.^23^ On an individual level, these strategies also enable students to identify and request additional resources they may need in a nonjudgmental environment. On a systemic level, a trauma‐informed approach mitigates the negative impact of systemic racism and other forms of discrimination in graduate education.^23^

**Table 3:** Challenges Among Boards Reboot & Readiness Students Related to National Certification Examination Preparation

Student‐Reported Challenges
Inadequate or undeused accommodations for learning differences or other disabilities
Lack of financial privilege to stop working during graduate school or examination preparation
Inadequate social support needed to study or provide empathy after feeling ashamed
Inadequate support to learn complex material at a fast pace (especially when English is not their primary or preferred language)
Inadequate support to learn test‐taking strategies (especially when English is not their primary or preferred language)
Lack of culturally congruent midwifery faculty
Isolation or lack of a consistent peer group
No external professional mentors

## COURSE EVALUATION AND LESSONS LEARNED

Faculty closely examine student feedback and NCE outcomes to make iterative revisions to the BRR course.^27^ Since launching the course in 2024, 28 students have completed it, which is defined as attending at least 5 of the 7 synchronous sessions and completing the mock examination. Of the 22 NM/WHNP students who completed the course and attempted the AMCB NCE, 91% successfully passed.

### BRR Course Improvement

Based on BRR student feedback via midterm and end‐of‐course evaluations, faculty revised some aspects of the fall and spring iterations. Students often begin the course hyperfocused on acquiring and completing MC practice questions. Many of them mistakenly rely solely on completing a large number of practice questions as their primary method of examination preparation. BRR faculty have increased their NCE preparation messaging that extensive question review alone is not associated with improved outcomes. Instead, faculty emphasize a more holistic approach, including a targeted content review via textbooks, other resources, and study groups. A second course improvement involved bolstering the virtual learning environment. Although faculty aimed to create a cohesive, brave, and safe virtual learning space, because students were from different cohorts, faculty made a greater effort to foster a sense of community and connection, emphasizing the importance of study groups through a shared contact list.

Faculty revised the weekly individualized study plan task to be completed over 3 weeks from 7. Students reported that by the third week, they had activated a study rhythm. Next, faculty noted that many students, particularly plurilingual students for whom English is not their primary language, struggled with “except” type questions (eg, “All of the following are a symptom of … *except*:…”), despite evident content mastery. Faculty removed these types of questions from course quizzes and examinations in 2023. However, because these questions persist on the AMCB and NCC examinations, they were reintroduced into the BRR course to familiarize students with this format. Finally, because the course content would be redundant, the faculty implemented a course policy limiting students from repeating the course if they took an NCE after the course and failed. Students also shared that repeating it felt defeating, adding to their stress and anxiety. In these rare occurrences, faculty suggested other board review options.

### Program Improvement

Although every student begins graduate nursing school with a bachelor's degree, not all have learned advanced study and test‐taking strategies. Faculty consistently noted that BRR students benefited from guidance on effective study techniques and time management tips. BRR students shared that they would have benefited from learning these strategies earlier in their program. As a result, the faculty added the week 1 strategies for success session to an introduction course for all NM/WHNP students.

## CONCLUSION

With AMCB pass rates declining since 2015 and the need for ACME‐accredited midwifery education programs to meet institutional pass rate goals, midwifery program faculty face challenges in supporting students who are at risk for failing their NCE(s). Graduates who do not pass face financial, emotional, and social burdens that can undermine their ability to pass in future attempts. Given the harms of systemic racism and other forms of bias and discrimination in midwifery education and the urgent need for more midwives, faculty and institutions must prioritize a trauma‐informed approach that promotes equity. One midwifery program has found an innovative, structured, and tuition‐free NCE preparation course to be an effective approach to increasing board pass rates. A year since its launch, through iterative improvements, the course has helped many at‐risk graduates restore their confidence, successfully pass their NCE, and enter the midwifery workforce with expediency and no additional financial burden.

## CONFLICT OF INTEREST

The authors have no conflicts of interest to disclose.
